# Chemical Characterization and *in Vitro* Cytotoxicity on Squamous Cell Carcinoma Cells of *Carica Papaya* Leaf Extracts

**DOI:** 10.3390/toxins8010007

**Published:** 2015-12-24

**Authors:** Thao T. Nguyen, Marie-Odile Parat, Mark P. Hodson, Jenny Pan, Paul N. Shaw, Amitha K. Hewavitharana

**Affiliations:** 1 School of Pharmacy, The University of Queensland, Brisbane, QLD 4072, Australia; t.nguyen65@uq.edu.au (T.T.N.); m.parat@pharmacy.uq.edu.au (M.-O.P.); m.hodson1@uq.edu.au (M.P.H.); jenny.pan@uqconnect.edu.au (J.P.); n.shaw@pharmacy.uq.edu.au (P.N.S.); 2 Metabolomics Australia, Australian Institute for Bioengineering and Nanotechnology, The University of Queensland, Brisbane, QLD 4072, Australia

**Keywords:** *Carica papaya*, cytotoxicity, mass spectrometry, cancer, flavonoids, chromatography

## Abstract

In traditional medicine, *Carica papaya* leaf has been used for a wide range of therapeutic applications including skin diseases and cancer. In this study, we investigated the *in vitro* cytotoxicity of aqueous and ethanolic extracts of *Carica papaya* leaves on the human oral squamous cell carcinoma SCC25 cell line in parallel with non-cancerous human keratinocyte HaCaT cells. Two out of four extracts showed a significantly selective effect towards the cancer cells and were found to contain high levels of phenolic and flavonoid compounds. The chromatographic and mass spectrometric profiles of the extracts obtained with Ultra High Performance Liquid Chromatography-Quadrupole Time of Flight-Mass Spectrometry were used to tentatively identify the bioactive compounds using comparative analysis. The principal compounds identified were flavonoids or flavonoid glycosides, particularly compounds from the kaempferol and quercetin families, of which several have previously been reported to possess anticancer activities. These results confirm that papaya leaf is a potential source of anticancer compounds and warrant further scientific investigation to validate the traditional use of papaya leaf to treat cancer.

## 1. Introduction

A book entitled “The most wonderful tree in the world—the papaw tree (*Carica papaia*)”, published some 100 years ago, contains many anecdotes relating to the cure of breast, liver or rectal cancer after “treatment” with *Carica papaya* preparations [1]. Subsequent reports have been published in various media that have detailed “the healing capabilities of an old Australian Aboriginal remedy—boiled extract of pawpaw leaves—against cancer” [2] and several other anecdotes relating “cancer cure” following consumption of various preparations of papaya plant [3,4,5,6].

Recently, we undertook a comprehensive literature review [7] and found that research providing scientific evidence for the effectiveness of *Carica papaya* in the treatment and prevention of cancer was limited. However, in contrast to the limited number of studies that have been done to evaluate the effects of papaya extracts on cancer, the abundance in *Carica papaya* of phytochemicals with reported anticancer activities, such as carotenoids (in fruits and seeds), alkaloids (in leaves), phenolics (in fruits, leaves, shoots) and glucosinolates (in seeds and fruits), suggests that there are opportunities for new research to evaluate the anticancer potential of this medicinal plant [7].

Squamous cell carcinoma (SCC) is the second most common type of skin cancer and also occurs in many other epithelia such as lips, mouth, urinary bladder, prostate, lung and vagina. Skin squamous cell carcinomas are not only more likely to metastasize but also to cause mortality, when compared with skin basal cell carcinoma [8]. Although different parts of the *Carica papaya* plant have been used as traditional medicine for the treatment of skin infections and wound healing in general, and this widespread use has been scientifically validated [9,10,11], no information is available on the activity of this plant on skin cancer. Furthermore, the effects of *Carica papaya* leaf extracts have previously been reported being tested on the growth of different cancer cell lines: breast, stomach, lung, pancreatic, colon, liver, ovarian, cervical, neuroblastoma, lymphoma, leukaemia and other blood cancers [12,13,14]; to our knowledge, no skin cancer cell lines have been tested. We hypothesized that *Carica papaya* leaf extracts exerted *in vitro* cytotoxicity on human squamous cell carcinoma. In this study, human oral squamous cell carcinoma (SCC25) cells and immortal, non-cancerous human keratinocyte cells (HaCaT) were selected for the cytotoxic studies of papaya extracts. The HaCaT cell line was selected to permit experiments to be performed in parallel with SCC25 in order to screen for candidate extracts with selective growth inhibition towards cancer cells, a highly desirable feature of potential cancer preventative and therapeutic agents. Our aim was also to preliminarily identify the bioactive compounds using liquid chromatography-quadrupole time-of-flight-mass spectrometry (LC-QToF-MS).

## 2. Results and Discussion

**Figure 1:**
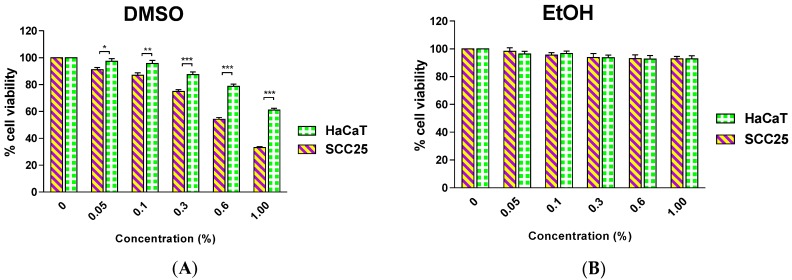
Effect of a 48-h incubation with Dimethyl sulfoxide (DMSO) (**A**); or Ethanol (EtOH) (**B**) on the survival of human squamous cell carcinoma (SCC25) and human keratinocyte (HaCaT) cells. Results are shown as mean ± SEM (*n* = 3). * *p* < 0.05, ** *p* < 0.01; *** *p* < 0.001, HaCaT *vs.* SCC25 (two-way analysis of variance (ANOVA) with Bonferroni post-tests).

The MTT assay has been widely applied in proliferation and cytotoxicity studies to screen the chemo-preventive potential of natural products. It provides preliminary data for further *in vitro* and *in vivo* studies. The addition of organic solvents is required to solubilize the extracts from natural products in cell culture media; therefore it is prudent to investigate the effect of the solvents on the cell lines under experimentation to identify the most suitable solvent and its optimal concentration in media. This information can then be used during sample preparation for rigorous cytotoxicity studies using the MTT assay. Dimethyl sulfoxide (DMSO) has been reported to be the solvent of choice for sample preparations with a final concentration in the medium from 0.1% to 1.0% but typically data have not been reported relating to impact of such DMSO concentrations on cell viability [15,16,17]. In our investigation, we found that DMSO at a concentration as low as 0.05% causes significant toxicity to SCC25 and a significantly different effect was observed between the two cell lines. In contrast, ethanol (EtOH) up to a concentration of 1.0% did not significantly impact upon the viability of either cell line (Figure 1).

**Figure 2:**
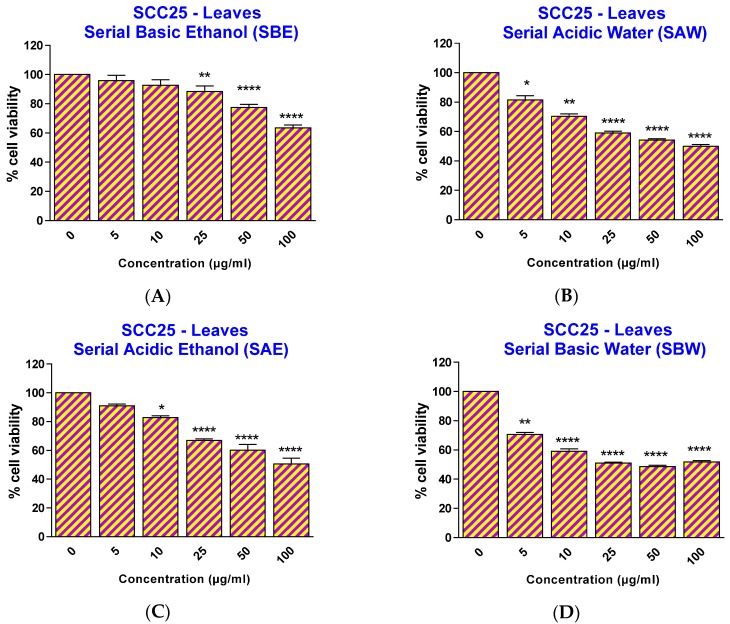
Effect of papaya leaf extracts (serially extracted in the listed order, with basic ethanol (**A**); acidic ethanol (**B**); acidic water (**C**); and basic water (**D**)) on the survival of SCC25 cells. Results are shown as mean ± SEM (*n* = 3). * *p* < 0.05; ** *p* < 0.01; **** *p* < 0.0001, *vs.* EtOH-treated control (one-way ANOVA with Kruskal-Wallis test).

**Figure 3:**
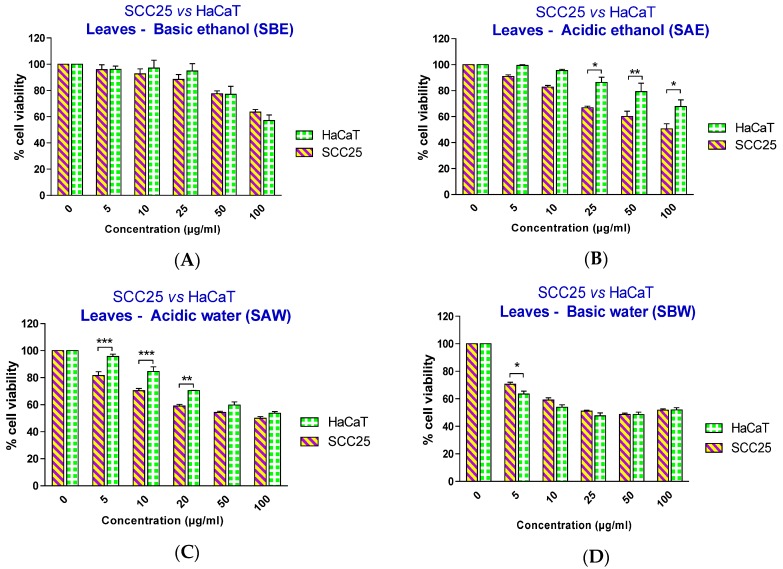
Effect of papaya leaf extracts (serially extracted in the listed order with basic ethanol (**A**); acidic ethanol (**B**); acidic water (**C**); and basic water (**D**)) on the survival of SCC25 and HaCaT cells. Results are shown as mean ± SEM (*n* = 3). * *p* < 0.05; ** *p* < 0.01; *** *p* < 0.001, HaCaT *vs.* SCC25 (two-way ANOVA with Bonferroni post-test).

Therefore, in this study, ethanol at a concentration of 0.3% was chosen as the solvent for the preparation of ethanolic extracts in media for cell experiments. To investigate the effect of the papaya leaf extracts on SCC25 and HaCaT cells, cells were treated with extracts over a range of concentrations (5–100 µg/mL) for 48 h and the percentage cell viability was analyzed. As revealed in Figure 2, all four extracts showed a significant effect on SCC25 cancer cell viability, starting at different concentrations: 25 µg/mL for serial basic ethanol (SBE), 10 µg/mL for serial acidic ethanol (SAE), and 5 µg/mL for both serial acidic water (SAW) and serial basic water (SBW) fractions. However, when the cell viability effects between SCC25 cancer cells and non-cancerous HaCaT cells were compared, the two fractions with acidic pH showed a significantly selective cytotoxicity towards the SCC25 cells with an effective range from 25–100 µg/mL for SAE and 5–20 µg/mL for SAW fractions (Figure 3).

The IC50 values clearly showed the selective effect of two acidic extracts with IC50 values for SCC25 cells smaller than thosefor HaCaT cells.This was not observed to be the case for either of the two basic extracts (Table 1). To eliminate the possibility that this selective effect might due to cancer cells being more sensitive to acidic pH than the non-cancerous cells, the pH of the medium containing extracts at the highest tested concentration (100 µg/mL) was measured. The pH of the media was unaffected by the addition of either acidic or basic extracts, likely due to the buffer capacity of the medium and the small addition of the extracts. Therefore, we conclude here that the acidic conditions provided extracts containing important compounds with selective effects on skin cancer cell viability.

**Table 1:** IC_50_ values of tested extracts for SCC25 and HaCaT cells.

Cell Lines	IC_50_ (µg/mL) (95% Confidence Interval)			
	Serial Basic Ethanol Extract	Serial Acidic Ethanol Extract	Serial Acidic Water Extract	Serial Basic Water Extract
SCC25	172.9	77.18	57.72	40.14
	(151.6–197.3)	(62.71–94.99)	(41.99–79.35)	(27.03–59.61)
HaCaT	157.6	199.5	85.74	34.24
	(119.8–207.4)	(155.4–256.2)	(71.63–102.6)	(21.47–54.60)

**Figure 4:**
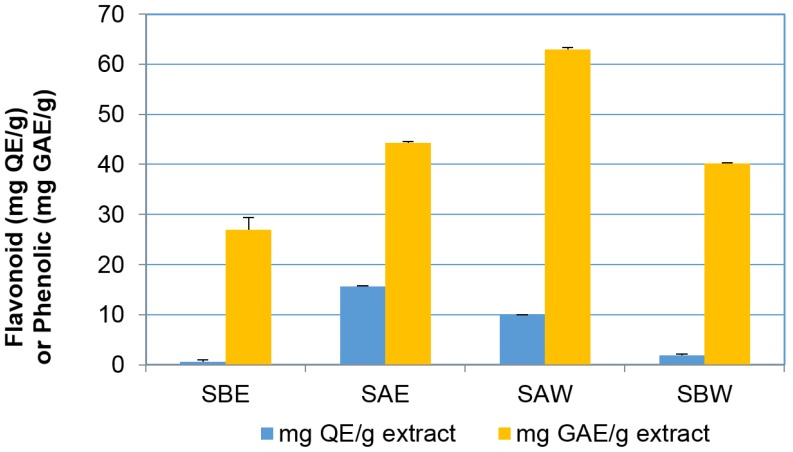
Total flavonoid and phenolic content in the extracts. Results are shown as mean ± SEM (*n* = 3).

Interestingly, examination of the phenolic and flavonoid content of the same four fractions (Figure 4) indicated that the phenolic and flavonoid contents positively correlated with the differential effect on cell viability as shown in Figure 3. Acidic ethanolic and water fractions had much higher flavonoid content (SAE = 15.60 ± 0.07; SAW = 9.95 ± 0.05 mg quercetin equivalents (QE)/g extract) compared to the basic fractions that contained less than 2.00 mg QE/g (SBE = 0.63 ± 0.30; SBW = 1.90 ± 0.23 mg QE/g extract). The phenolic content of acidic fractions was also found to be higher than that in basic fractions but to a lesser extent than the flavonoid content (SAW = 62.98 ± 0.30; SAE = 44.26 ± 0.27 mg gallic acid equivalents (GAE)/g extract and SBW = 40.18 ± 0.16; SBE = 26.92 ± 2.53 mg GAE/g extract).

**Scheme 1:**
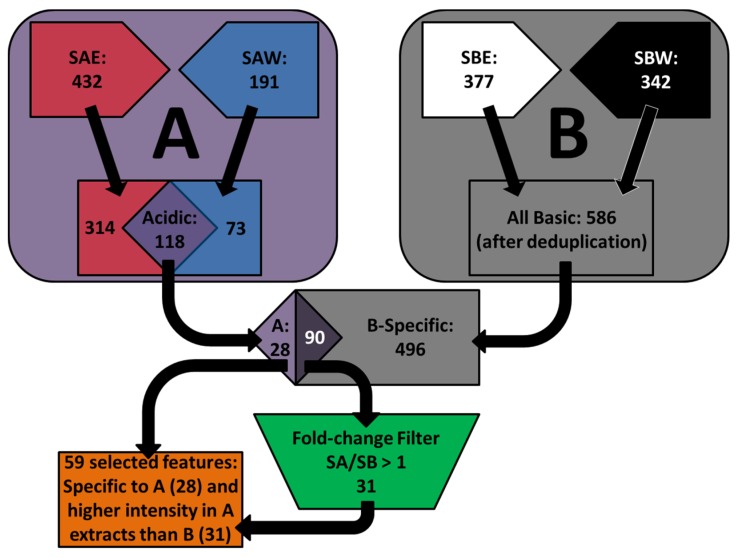
Diagrammatic representation of the selection of features obtained from comparative analysis of chromatographic profiles between acidic and basic extracts in positive ionization mode ((**A**) Acidic extracts; (**B**) Basic extracts).

The apparent positive correlation between the selective effects on the SCC25 cancer cells and the content of flavonoids and phenolic compounds initiated further comparative analysis of the chemical constituents to identify the compounds that are present exclusively or at higher concentrations in the acidic extracts compared to basic extracts. The chromatographic data were extracted to features by Molecular Feature Extractor algorithms and then aligned by Mass Profiler Professional software (Version 12.1, Agilent Technologies, Santa Clara, CA, USA, 2012). In positive ionization mode, a total of 432 and 191 features were detected in acidic water and acidic ethanolic extracts, respectively; 118 features were common to both extracts. These 118 features were compared to the 586 features that appeared in either of the basic extracts in order to search for the features detected specifically in acidic extracts or with higher intensity than in basic extracts. A total of 59 features were found to fit these criteria (Scheme 1).

A similar procedure was then applied to the features obtained in negative ionization mode, resulting in five features of interest. The neutral mass of the 64 features obtained in both modes was queried against the METLIN Personal Metabolite Database (https://metlin.scripps.edu) and the customized NPACT database (http://crdd.osdd.net/raghava/npact) to match to compounds using a mass tolerance window of ≤10 ppm. The METLIN database contains 64,092 structures of endogenous and exogenous metabolites (as of June 2015) whereas the customized NPACT database consists of 1,574 entries of plant-derived natural compounds that exhibit anti-cancerous activity [18]. The METLIN-based search resulted in several candidates (a total of 880 hits for 64 features) and directed further investigation with MS fragmentation spectra to confirm the identities of bioactive compounds in the extracts (Table 2).

**Table 2:** Tentative identification of compounds which appear in acidic extracts exclusively or at greater extent than in basic extracts.

Formula	Experimental Mass	Error (ppm) *	Number of Candidates from Metlin Database	Putative Compounds from NPACT Database
C_4_H_5_NO_3_	115.0269	0	1	-
C_5_H_7_NO_3_	129.0428	1	7	-
C_14_H_25_NO_3_	255.1835	0	1	-
C_14_H_25_NO_3_	255.1846	4	1	-
C_15_H_10_O_6_	286.0486	3	28	Fisetin, Kaempferol, Luteolin, Scullarein, Tetrahydroxyflavone
C_18_H_33_NO_2_	295.252	2	1	-
C_15_H_10_O_7_	302.0436	3	22	Quercetin; Morin;Viscidulin I
C_18_H_19_NO_3_	307.2158	3	4	-
C_22_H_43_NO	337.335	1	3	-
C_22_H_41_NO_2_	351.3145	2	1	-
C_21_H_30_O_10_	442.1843	0	1	-
C_21_H_20_O_11_	448.1009	0	74	Kaempferol β-d-glucopyranoside, Luteolin β-d-glucopyranoside
C_21_H_20_O_12_	464.0958	0	56	Myricetin 3-*O*-rhamnoside
C_20_H_26_O_13_	474.1373	0	1	-
C_28_H_46_N_2_O_4_	474.346	0	1	-
C_28_H_46_N_2_O_4_	474.3467	1	1	-
C_28_H_46_N_2_O_4_	474.3468	2	1	-
C_28_H_48_N_2_O_4_	476.3624	2	1	-
C_30_H_48_N_2_O_4_	500.3617	0	1	-
C_26_H_55_O_8_P	526.3631	0	1	-
C_33_H_50_O_5_	526.3631	5	1	-
C_34_H_56_N_2_O_4_	556.4245	0	1	-
C_29_H_44_O_12_	584.2809	4	2	-
C_34_H_48_O_8_	584.3322	4	3	-
C_27_H_30_O_15_	594.158	0	120	-
C_27_H_30_O_16_	610.153	0	109	Rutin
C_27_H_30_O_16_	610.1531	0	109	-
C_28_H_32_O_16_	624.1686	0	78	-
C_28_H_32_O_16_	624.1689	0	78	-
C_34_H_65_O_13_P	712.4155	1	1	-
C_33_H_40_O_19_	740.2154	1	33	-
C_33_H_40_O_20_	756.2107	0	81	-
C_33_H_40_O_21_	772.2069	0	56	-
C_46_H_77_O_9_P	804.5319	1	1	-

* Error (ppm): the difference between experimental mass and theoretical mass of compound.

Among the tentatively identified metabolites from the METLIN database, the most remarkable were the flavonoids or flavonoid glycosides (kaempferol, quercetin, rutin, manghaslin, nicotiflorin, clitorin, quercetin 7-galactoside, myricetin 7-rhamnoside, luteolin 3,7-diglucoside). These correlated with the results of the total flavonoid content in acidic extracts compared to basic extracts. Furthermore, the search within NPACT database revealed several compounds with previously reported anti-cancer activities including fisetin, kaempferol, luteolin, scullarein, tetrahydroxyflavone, quercetin, morin, viscidulin I, kaempferol β-D-glucopyranoside, luteolin β-d-glucopyranoside, myricetin 3-*O*-rhamnoside, and rutin [18]. Kaempferol and quercetin have been detected and quantified in *Carica papaya* leaves by gas chromatography-mass spectrometry analysis with quantities of 0.03 ± 0.001 mg/g and 0.04 ± 0.001 mg/g dry leaf, respectively [19]. Due to the presence of aromatic phenol groups, these flavonoids are considered weak acids; therefore they would be more readily extracted into an acidic environment in preference to a basic environment. Quercetin has been found to selectively affect the viability of SCC25 cells without causing toxicity to human gingival fibroblasts [20]. The mechanism by which quercetin inhibited the proliferation of SCC25 cells included both G1 phase cell cycle arrest and mitochondria-mediated apoptosis. Quercetin further decreased the migration and invasion of SCC25 cells in a dose-dependent manner [20]. For other flavonoids, many mechanisms of action on tumours have been identified such as inhibition of proliferation, apoptosis induction, carcinogen inactivation, impairment of invasion and angiogenesis [21,22]. The molecular mechanisms by which flavonoids exert these effects have been proposed to include the signalling pathways of PI3-kinase (phosphoinositide 3-kinase), Akt/PKB (protein-kinase B), tyrosine kinase P1KC (protein-1 kinase C) and MAP (mitogen-activated protein) kinase as reviewed elsewhere [23]. Using primary SCC cells and normal oral mucosa cells as a control, the flavonoid morin was shown to cause G2/M arrest without causing apoptosis, and to impact kinases AKT, JNK and p38 signaling pathways [24]. However, of the three kinases, AKT was the only one that was selectively inhibited in cancer cells compared to non-cancer cells, and the authors suggested that AKT might mediate the enhanced tumour sensitivity to morin [24].

Although our study presents limitations inherent to this type of research, in which database searching and matching based on accurate mass data alone provides numerous compound identities for each mass, the results revealed that papaya leaf acidic extracts contain numerous bioactive compounds with selective activities on SCC cells. Further studies are required to confirm the identities of these compounds by wider variety of isolation, purification and identification techniques (MS^n^, NMR) and to investigate the possible mechanism of anticancer activities of *Carica papaya* leaf extracts.

## 3. Experimental Section

### 3.1. Chemicals and Reagents

Dulbecco’s Modified Eagle’s Medium (DMEM), DMEM-F12, penicillin/ streptomycin, trypsin, foetal bovine serum (FBS) were purchased from Invitrogen (Life Technologies, Mulgrave, VIC, Australia). 3-(4,5-Dimethylthiazol-2-yl)-2,5-diphenyltetrazolium bromide (MTT), dimethyl sulfoxide (DMSO), LC-MS grade ammonium bicarbonate and formic acid, Folin-Ciocalteu’s phenol reagent, gallic acid (>97.5% purity), quercetin (98% purity) and epigallocatechin-3-gallate (EGCG) (99%) were obtained from Sigma-Aldrich (Castle Hill, NSW, Australia). HPLC grade methanol and acetonitrile were obtained from Merck (Darmstadt, Germany). Other chemicals such as hydrochloric acid, sodium hydroxide were of analytical grade purchased from Ajax Finechem (Cheltenham, VIC, Australia). Purified water was generated using a Milli-Q system (Millipore, Billerica, MA, USA).

### 3.2. Preparation of Papaya Leaf Extracts

Fresh *Carica papaya* leaves were collected from Tropical Fruit World (TFW), a privately-owned plantation orchard farm and research park in northern New South Wales, Australia (http://www.tropicalfruitworld.com.au/). Permission for the use of the *Carica papaya* leaves was granted by Aymon Gow, manager of TFW. The papaya plants in this facility are neither protected nor endangered species and had not been sprayed with any chemicals. The leaves were thoroughly washed under running tap water to remove any particulate matter, and then rinsed with deionized water to obtain clean leaves. A Christ Alpha 2-4 LD freeze-dryer was used to lyophilize the leaves at −60 °C and 0.1 mbar, for 24 h. Dried leaves were then ground into powder using a food processor (Oskar Mini, Sunbeam, NSW, Australia). The dried powder was portioned and stored at −80 °C until extraction.

A mass of 20 g of the freeze-dried leaf powder was extracted sequentially with ethanol and water in acidic or basic conditions in the order: basic ethanol, acidic ethanol, acidic water, basic water (2 × 200 mL for each solvent; pH was maintained at pH 1–2 or 10–11 during the extraction process by adjusting with either 10% hydrochloric acid or 5 M sodium hydroxide solution). The obtained ethanolic extracts were concentrated at 30 °C and 120 rpm under vacuum by a rotary evaporator (IKA^®^RV 10, IKA-Werke, Staufen im Breisgau, Germany). The resulting concentrates of ethanolic extracts and water extracts were lyophilised using the freeze-dryer. All lyophilised fractions (Basic ethanol: SBE, Acidic ethanol: SAE, Acidic water: SAW, Basic water: SBW) were stored at −80 °C prior to analysis.

### 3.3. Cell Culture Conditions

SCC25 cells (ATCC® CRL-1628™, Manassas, VA, USA) were maintained in DMEM/F12 medium supplemented with 10% *v*/*v* heat-inactivated foetal bovine serum, 1% penicillin-streptomycin and 0.4 μg/mL hydrocortisone. HaCaT cells (a generous gift from Professor Fusenig) [25] were propagated in DMEM medium supplemented with 10% foetal bovine serum and 1% penicillin-streptomycin. The cells were grown in a humidified incubator at 37 °C in a 5% CO_2_ atmosphere. Cells were passaged every 3 days and cultures were allowed to reach 70%–90% confluence before experiments were performed.

### 3.4. Cell Viability Assays

The effect of extracts on SCC25 and HaCaT viability was evaluated using the colorimetric tetrazolium dye procedure commonly referred to as the 3-(4,5-dimethylthiazol-2-yl)-2,5-diphenyltetrazolium bromide (MTT) assay developed by Mosmann with minor modification [26]. SCC25 or HaCaT cells were plated into 96-well plates at densities of 6 × 10^3^ cells per well in 100 µL of DMEM/F12 (10% serum) and 3 × 10^3^ cells well in 100 µL of DMEM (10% serum), respectively. Cells were incubated at 37 °C for 24 h and were subsequently treated for 48 h with 0.5% serum medium containing increasing concentrations (5–100 µg dry mass/mL) of extracts in ethanol (0.3% final concentration in medium). Control cells were exposed to an equivalent volume of ethanol (0.3% final concentration). Cells were then incubated in 100 µL of MTT-containing medium (0.2 mg/mL MTT in 0.5% serum medium) at 37 °C for an additional two hours. The medium was then removed and the formazan crystals trapped in cells were dissolved in 100 µL of DMSO by gentle shaking for 20 min on an orbital shaker. Absorbance of the solubilized product was measured at 595 nm using an Imark plate reader (BioRad, Hercules, CA, USA). The absorbance of cells exposed to medium containing 0.3% ethanol only was taken as 100% cell viability (*i.e.*, the control). The results are expressed as percent of the viability of control cells ± standard error of the mean (SEM) from 4–8 parallel determinations in three independent experiments (*n* = 3). Dose-effect analysis on SCC25 cells was performed by one-way analysis of variance (ANOVA) with Kruskal-Wallis test (as the data were non-parametric). Differences between the SCC25 and HaCaT cell lines, and interaction between cell line and extract effects were analysed by two-way ANOVA with Bonferroni post-tests. All statistical analyses were carried out using GraphPad Prism 6.0 (GraphPad Software Inc., San Diego, CA, USA, 2014).

### 3.5. Determination of Total Phenolic Content

Total phenolic content of ethanol and water extracts was determined using the Folin-Ciocalteu assay as described previously [27] with minor modifications. Briefly, 0.5 mL of diluted extract was mixed with 2.5 mL of freshly prepared Folin-Ciocalteu’s phenol reagent, followed by the addition of 2 mL of 7.5% Na_2_CO_3_. The mixture was vortex mixed for 2 min and left in the dark at room temperature for 30 min. The absorbance was then measured at the maximum wavelength of 758 nm against a blank comprising 0.5 mL diluted extract, 2.5 mL water and 2 mL of 7.5% Na_2_CO_3_. Gallic acid was used as the standard, and results were expressed as gallic acid equivalents (GAE) in mg/g of dry weight of each extract.

### 3.6. Determination of Total Flavonoid Content

Total flavonoid content of the extracts was determined using a colorimetric assay developed previously [27]. Diluted extract was mixed with 2% AlCl_3_ in ethanol in equal volume and absorbance was measured after 15 min at 425 nm, against the blank sample consisting of equal volume of dilute extract and ethanol without AlCl_3_. Quercetin was used as the standard, and results were expressed as quercetin equivalents (QE) in mg/g of the dry weight of each extract.

### 3.7. UHPLC-ToF-MS Analysis

Chromatographic analysis of compounds in papaya leaf extracts was performed on an Agilent 1290 UHPLC system (Agilent Technologies, Santa Clara, CA, USA). Chromatographic separation was achieved on a 2.1 × 150 mm, 3.5 μm ECLIPSE PLUS C18 analytical column (Agilent) with guard protection. Mobile phase A was purified water containing 5 mM ammonium bicarbonate and 0.1% formic acid, adjusted to pH 7.0 ± 0.1 and mobile phase B was 95% acetonitrile and 5% water (*v/v*) containing 5 mM ammonium bicarbonate and 0.1% formic acid. The following gradient elution was adopted: 10% to 80% B for the first 42 min; 80% to 90% B from 42 to 45 min; 90% to 100% B from 46 to 48 min; held at 100% B from 48 to 50 min; returned to 10% B over the next 2 min, and the column re-equilibrated with 10% B for 10 min prior to the next injection. Thus the total chromatographic run time was 60 min. A flow rate of 0.2 mL/min was applied and 20 μL of sample was injected.

Each extract was run in triplicate in both positive and negative ionization mode. Mass spectrometric detection was performed on an Agilent 6520 high-resolution accurate mass quadrupole time-of-flight (Q-ToF) mass spectrometer equipped with a multimode source in both Electrospray Ionisation (ESI) and Atmospheric Pressure Chemical Ionization (APCI). Mass spectra were controlled using MassHunter acquisition software (Version B.02.01 SP3, Agilent Technologies, Santa Clara, CA, USA, 2010). The mass spectrometer was operated in the range of *m/z* 100–1700, at a scan rate of 0.8 cycles/second under the following conditions: capillary voltage 2500 V, nebulizer pressure 30 psi, drying gas flow 5.0 L/min, gas temperature 300 °C, fragmenting voltage 175 V, skimmer voltage 65 V. To ensure the desired mass accuracy of recorded ions, continuous internal calibration was performed during analysis with the use of reference ions—*m/z* 121.050873 (protonated purine) and *m/z* 922.009798 (protonated hexakis) in positive mode; in negative mode, ions with *m/z* 119.0362 (deprotonated purine) and *m/z* 966.000725 (formate adduct of hexakis) were used to correct for scan to scan variations.

### 3.8. MS Data Analysis

Data analysis was performed using Agilent MassHunter Qualitative software (Version B.05.00, Agilent Technologies, Santa Clara, CA, USA, 2012) with Molecular Feature Extractor (MFE) algorithms in concert with Mass Profiler Professional software (Version 12.1, Agilent Technologies, Santa Clara, CA, USA, 2012) to align features from the chromatograms of all samples from four extracts of papaya leaves. The following cut-off settings were employed: minimum peak filters of 500 counts, peak spacing tolerance of 0.0025 *m*/*z* plus 7.0 ppm, assigned charge states limited to a maximum of two, minimum compound filters of 3000 counts. The Molecular Feature Generator algorithm was utilised to generate putative molecular formulae from the following common elements C, H, N, O, P and S. Compound identification was carried out by using a Personal Compound Database Library (PCDL) (Agilent, Santa Clara, CA, USA) with the METLIN Personal Metabolite Database and a customized PCDL database which was compiled using the PCDL platform with compounds obtained from Naturally occurring Plant-based Anticancerous Compound-Activity-Target Database (NPACT) [18].
